# Kampo Therapies and the Use of Herbal Medicines in the Dentistry in Japan

**DOI:** 10.3390/medicines6010034

**Published:** 2019-02-28

**Authors:** Shuji Watanabe, Toshizo Toyama, Takenori Sato, Mitsuo Suzuki, Akira Morozumi, Hiroshi Sakagami, Nobushiro Hamada

**Affiliations:** 1 Division of Microbiology, Department of Oral Science, Kanagawa Dental University, 82 Inaoka-cho, Yokosuka 238-8580, Japan; swatanabe2010@nifty.com (S.W.); toyama@kdu.ac.jp (T.T.); t.sato@kdu.ac.jp (T.S.); suzuki@d2clinic.jp (M.S.); 2 Odoriba Medical Center, Totsuka Green Dental Clinic, 1-10-46 Gumizawa, Totsuka-ku, Yokohama 245-0061, Japan; 3 Dental Design Clinic, 3-7-10 Kita-aoyama, Minato-ku, Tokyo 107-0061, Japan; 4 Morozumi Dental Clinic, 1-3-1 Miyamaedaira, Miyamae-ku, Kawasaki 216-0006, Japan; morozumident@gmail.com; 5 Meikai University Research Institute of Odontology (M-RIO), 1-1 Keyakidai, Sakado, Saitama 350-0283, Japan; sakagami@dent.meikai.ac.jp

**Keywords:** periodontitis, Kampo, traditional medicine, *Jixueteng*, *Juzentaihoto*, technical terms, gargle, tongue diagnosis, mastic, pathogenic factors

## Abstract

Dental caries and periodontal disease are two major diseases in the dentistry. As the society is aging, their pathological meaning has been changing. An increasing number of patients are displaying symptoms of systemic disease and so we need to pay more attention to immunologic aggression in our medical treatment. For this reason, we focused on natural products. Kampo consists of natural herbs—roots and barks—and has more than 3000 years of history. It was originated in China as traditional medicine and introduced to Japan. Over the years, Kampo medicine in Japan has been formulated in a way to suit Japan’s natural features and ethnic characteristics. Based on this traditional Japanese Kampo medicine, we have manufactured a Kampo gargle and Mastic Gel dentifrice. In order to practically utilize the effectiveness of mastic, we have developed a dentifrice (product name: IMPLA CARE) and treated implant periodontitis and severe periodontitis.

## 1. Introduction

Kampo medicine in Japan is the Oriental medicine which was originally brought from China through east Asia and the Korean peninsula as the ancient Chinese medicine. During the Nara period of Japan (AD 710-784), Japanese envoys were sent to Tang Dynasty (AD616-907) under the name of Kentoshi and it became a channel that Chinese medicine was directly brought to Japan. In the Kamakura period (AD1185-1333), it made a progress to become more practical medicine by being adopted more to Japan’s natural environment, rather than a simple copy of Chinese medicine. In the Muromachi period (AD 1336-1573), a Japanese whose name was Sanki Tashiro studied in China during its Ming dynasty (AD1368-1644) and created the academic foundation of the Japanese Kampo medicine. Kampo was first used in higher social classes but since the 15th century it has provided general people natural material-based medicine. In the Edo period (AD 1603-1867), Western medicine was introduced to Japan from Netherland and was called Rangaku, innovating special characteristics such as abdominal diagnosis and contributed to the developments of medical diagnosis and treatment [1,2,3]. On the other hand, the traditional medicine which was primarily using herbal medicines was called Kampo medicine. In 1883, the Japanese government promulgated the regulation of the medical license that only those doctors who have mastered the Western medicine could prescribe the Kampo medicines and the Kampo medicine declined since then except for the practice by some facilities or individuals. In 1927, the book of Kokan Igaku (traditional Japanese and Chinese medicine) by Kyushin Yumoto was published and it triggered the subsequent revival of Kampo medicine. Following that in 1950, the academic society of “Japan Society of Oriental Medicine” of Kokan Igaku was established, which became the association of Oriental medicine including acupuncture and moxibustion treatments. Under the guidance of the Japan Medical Association, Kitasato University Oriental Medicine Research Centre was founded in 1972 and it has played a central role in the education of Oriental Medicine. Further in 2001, the model curriculum of medical education newly incorporated a course of study for “being capable of explaining Japanese and Chinese medicines.” At present, all medical schools in Japan have the courses of study for Kampo medicine or Oriental medicine as part of their education program.

Kampo medicine has a unique character, which is different from Western medicine. Kampo uses as a combination drug of various herbal plants that have complementary physiological activities. In fact, 148 Kampo formulations are used for medicinal treatments and are covered by the Japanese National Health Insurance Program [4]. Since 2012, seven kinds of Kampo formulations were approved by Japan Dental Association within the National Health Insurance Drug Price Standard related with dental treatment [5]. In 2015, Kampo Education Plan of Dentistry was sent from the Japan Dental Association to all dental universities [6]. At the same time, the first author of this article (S.W.) established the Yokohama Kampo Dentistry Study Group for the continuing education of Kampo. This group has held 18 research sessions and symposiums by calling a special lecturer of Kampo and is planning to publish a side reader of 11 Kampo preparations for dental students from the Nanzando publishing company. There are two academic societies on Kampo in Japan: The Japan Society for Oriental Medicine and Japan Dental Society of Oriental Medicine. The latter showed previously different direction about *qigong* and massage but now changing to the same direction with the former. 

Kampo prescriptions in dentistry has special characteristics due to the diverse symptoms such as the pain caused by the bad bite alignment or malocclusion (exogenous cause) and unidentified complaints. For Kampo prescriptions, it is important to understand the symptoms, by considering these special characteristics. It has also become known that the negative feedback functionality would not work when malocclusion and occlusal destruction become chronic with the sympathetic-nerve predominant state. We expect that Western medicine and the Oriental medicine will be used selectively and in parallel in many of the clinical cases in dentistry to ensure the best results for primary cares and that it would contribute to the dentistry medicine going forward.

In this review article, we firstly focus on the basic theory of Kampo medicine and then its biological activities and clinical effects in the dental treatments.

## 2. Basic Theory of Kampo Medicine

### 2.1. Concept of Oriental Medicine

**Figure 1:**
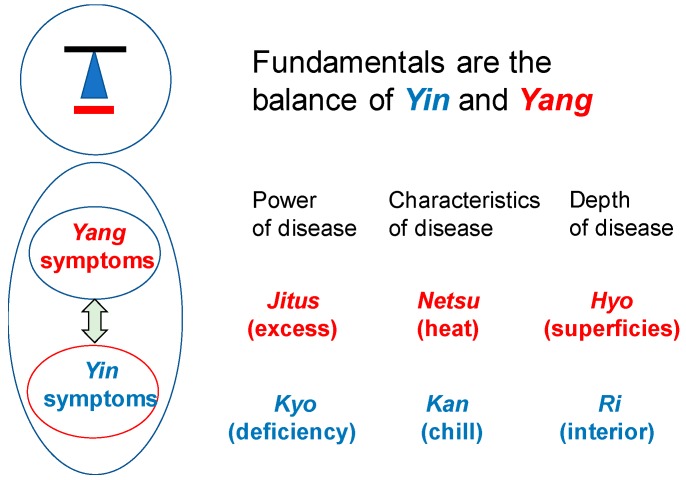
Concepts of Kampo therapy. Kampo therapy begins with the knowledge of constitutional characteristics of patient’s body. *In-Yo* or *Yin-Yang*: When the switch-over between two representative autonomic nerves, the sympathetic nerves (*yang)* and the parasympathetic nerves *(yin*), is good, the *yin-yang* balance is kept well. *Kyo-Jits* or asthenia and sthenia show physical strength, constitutional characteristics of body and the strength of resistance against disease. The reaction differs, depending on their *Kyo-Sho* or *Jitsu-Sho.* It is categorized into *Jitsu-Sho* (excess symptom)*, Kyo-Sho* (deficiency symptom) and *Chukan-Sho* (symptom in-between the two). *Kan-Netsu* or chills and fever: *Kan* is *Yin* while *Netsu* is *Yang.* They are always in a relative relation. When *Yin* deteriorates, *Yang* predominates, called *Netsu-sho* (heat syndrome).

Western medicine is a proof medicine that is based on the medical evidence, while Oriental medicine is a traditional medicine that has accumulated the evidence based on the experiences [7]. The modern medicine has made great advances by accumulating scientific evidences but it was at the same time a challenge to the limit of the medicine and it has added a new dimension of problems such as the side effects of medicines to cope up with the aggravation of diseases. In recent years, medical evidences of Kampo prescription (traditional medicine in Japan based on the traditional Chinese medicine), which is one of the pharmacotherapies of Oriental medicine, has become widely known. It is making it possible to include Kampo in the pharmacotherapy of the modern medicine, in consideration of its role as biological response modifiers (BRMs) of the vital balance. Oriental medicine was generated from the concept that the natural world consists of the opposing axis of *Yin* (Table A1) or “non-resistant” and *Yang* or “resistant,” where the relativity of these axes maintains the qualitative balance of the workings of mother nature [3]. This principle is therefore called the “*Yin*-*Yang* theory.” In the Kampo therapy, the body state and each internal organ are divided into *Yin* or *Yang* and it treats the patient so the *yin*-*yang* is balanced and can maintain the homeostasis (Figure 1).

### 2.2. Differences from the Western Medicine

Oriental medicine and Western medicine are both medical sciences, fundamentally and equally. Oriental medicine identifies the disease mainly based on the pathological condition at the time of diagnosis. It understands that a part of the body appeals the problem and it spreads to the entire body and shows the symptom in that part. Therefore, the treatment aims at alleviating the symptom while improving the pathological condition itself. This pathological condition is called *Sho* or Kampo Diagnosis. 

**Figure 2:**
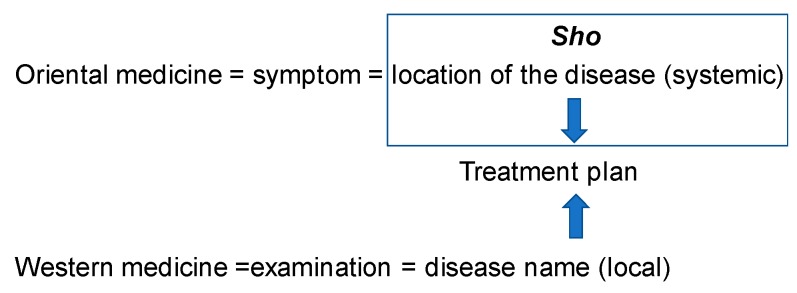
Characteristics of Oriental medicine and Western medicine therapy. *Sho* (Kampo Diagnosis): Capturing the *Shoko* (*yin-yang*, *Kyo-Jitsu* or deficiency and excess, *Kan-Netsu* or chills and fever, *Hyo-Ri* or superficies and interior) as the holistic symptom caused by the pathological condition and it shows the condition of the patient at the time of diagnosis. *Sho* changes depending on the bodily sensation.

The Western medicine diagnoses based on the demonstrated symptom and it emphasizes the importance of the examinations in order to find out the condition of the appearing symptoms, then gives it a name and treats it accordingly. On the other hand, Kampo medicine improves the symptom by improving the pathological condition presented in the form of *Sho* [2,8]. *Sho* is the measure to know the characteristics, size and depth of the disease, which would indicate the cause of the disease and the treatment, is given by finding out the cause of the pathological condition by *Sho*. From this, Oriental medicine makes it possible to treat the patient as expressed in the phrase “Different treatments for one disease; identical treatment for different diseases.” It means that different treatments will be given for the same disease name of patients if the *Sho* (Kampo diagnosis) is different. Likewise, the same treatment will be given to the different disease names of patients, if the *Sho* is the same. Here in this article, the disease name means the name of the disease under the modern medical science (Figure 2). 

### 2.3. The Categories of Sho

**Figure 3:**
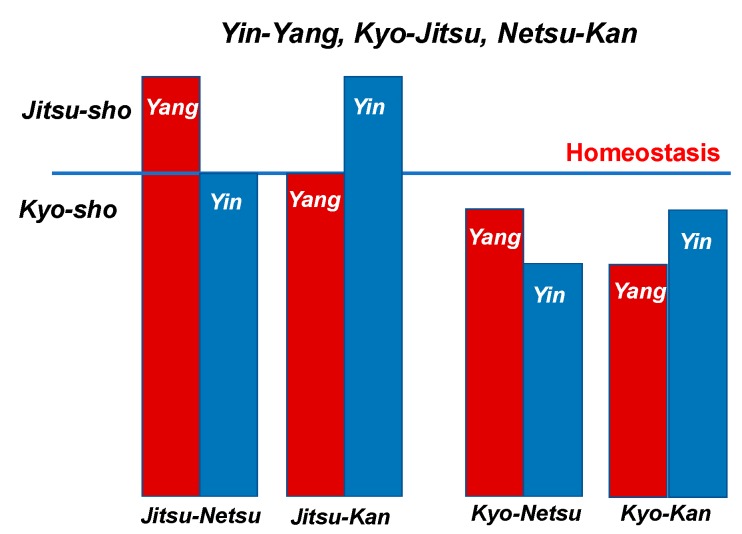
Condition categories of *Yin-Yang*, *Kyo-sho* and *Netsu-Kan* in the body.

*Shoko* (Kampo medical conditions, symptoms in Western medical terms) consists of *Yin-*Yang with *Kyo-Jitsu*, *Kan-Netsu* and *Hyo-Ri*. They reflect the current pathogenic condition. Table 1 shows the definitions and classifications of *Yin*-*Yang*, *Kyo-Jitsu* (asthenia and sthenia) and *Kan Netsu* (chills and fever). Table 2 shows the definitions of *Yin-Kyo*, *Yo*-*Kyo*, *Yin*-*Jitsu* and *Yo*-*Jitsu*. Figure 3 shows the condition categories of *Yin*-*Yang*, *Kyo*-*sho* and *Netsu*-*Kan* in the body. 

On the other hand, *Hyo-Ri* (superficies and interior) indicates where the disease exists in the body and shows the depth of *Sho,* depending on the part of the body. *Hyo*-*ri* is a unique concept of Kampo medicine, which indicates the seriousness of the disease, based on the location and depth of the disease. According to the classic literature “Shokan-ron,” *Yin* and *Yang* are further divided into 3 layers by the degree of development and it calls them the “Three *Yin*” and the “Three *Yang*” of diseases [8]. The Three *Yang* stage indicates *Hyo* or the surface of the body such as skin but also includes the parts of the body that show the *Hyo* symptoms such as chill, fever, headache, joint pain and sweat. The three *Yin* stage indicates *Ri*. Mostly *Sho*’s of the internal organs, including heart, lungs and digestive apparatus, are shown, where the symptoms are, for example, abdominal pain, constipation, diarrhoea, fullness of the abdomen, stomach tension. The middle part between the *Hyo*-*Ri* is called *Han-Hyo-Ri* or mesodermal.

### 2.4. Cause of Illness

Disease in Kampo medicine is the disorder of harmony of spirit and body. The living body has physiological functions to maintain the homeostasis of the organism against the changes of internal and external environment. This function is called *Shoki* or healthy *Ki. Shoki* is a function which would defend the body against disease and would induce natural healing ability, where the natural healing ability would seek for the harmony of *Ki, Ketsu and Sui* (vital energy, blood circulation and aqua). In contrast, the factor that attempts to destroy the homeostasis is called *Ja* or *Byoja* or stress or pathogen. It induces the climate, emotion or virus to cause various physical abnormalities which would lead to diseases. Causes of disease are categorized into endogenous, exogenous and other factors.

Endogenous factor means that the cause of disease comes from the inside of body. It may be common emotions of human beings. It is a physiological phenomenon and may be quintessential in life. However, it can cause abnormality in the functions of internal organs, when mental stress persists or when a sudden strong shock hits us, because it would collapse the balance of *Yin*, *Yang*, *Ki* and *Ketsu* on such occasions. It is called the *Seven Emotions* –joy, anger, anxiety, worry, grief, apprehension and fear.

Exogenous factor means that *ja* invades the body from outside. Diseases occur influenced by the mental or physical changes when the body cannot accept the natural environment such as pathogens, severe natural environment and sudden climate changes. It is called the *Six Pathogenic Agents* - wind, cold, heat, humidity, dryness and fire (fever). 

These are other factors, which would cause diseases due to other causes such as inadequate eating-drinking, fatigue, overwork, injury, poisoning, parasite and heredity.

### 2.5. Explanation of Ki, Ketsu, Oketsu and Sui

**Figure 4:**
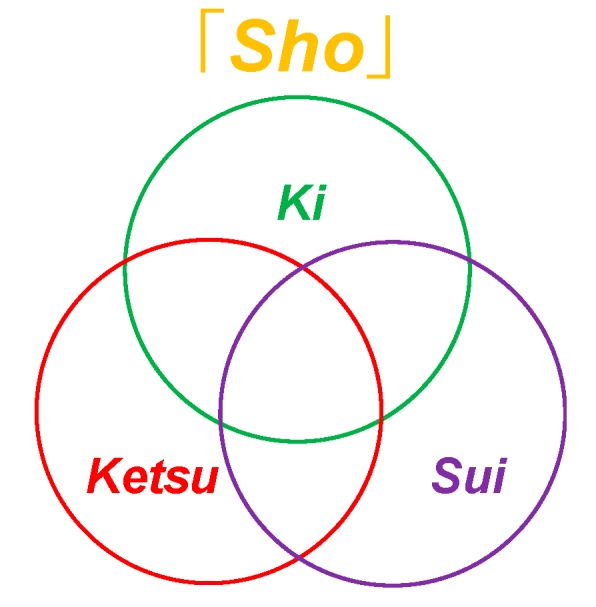
Diagrams of biological factors in *Sho*.

The concept of *Ki* (vital energy), *Ketsu* (blood/blood circulatory function) and *Sui* (aqua) is the fundamental concept to understand the Kampo medicine [2,8] (Figure 4). 

*Ki* indicates *yang* and *Ketsu* and *Sui* indicate *yin*. Patients reversibly show *Jitsu*-*Sho* (excessive) and Kyo-Sho (deficiency) (Table 3).

#### 2.5.1. *Ki*, Vital Energy

*Ki* is the source of energy of the entire biological activities and it circulates blood and bodily fluid in the whole body [8]. Therefore, the inhibition of the workings of *Ki* would inhibit the workings of *Ketsu* (blood circulation) and *Sui* (bodily liquid) and thus a disease would occur. *Ki* belongs to *yang*. When these elements are excessively active, it is called *Jitsu* or excess and is called *Kyo* or deficiency when these are deficient. Excessive *Ki* is called *Ki-Gyaku* (*ki-Jitsu*) or hyperactive *ki*. It is called *Ki-Tai* when stagnated due to over excess, while *Ki-Kyo* when under hypersecretion. 

#### 2.5.2. *Ketsu*, Blood Circulation

**Figure 5:**
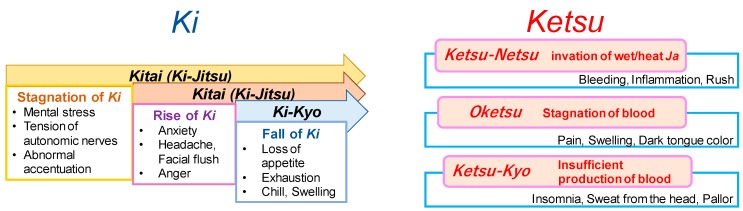
The concepts of *Ki* and *Ketsu*.

Blood circulates through the body and supplies nutrition to the five parenchymatous viscera (heart, liver, spleen, lung and kidney) and the six hollow viscera [gallbladder, stomach, small intestine, large intestine, urinary bladder and triple heater (*sansho*, a passage that controls the flow of air, blood and water)]. *Ki* and *ketsu* are interdependent each other –*Ki* warms up the body in *Yang and ketsu* nourishes the body in *Yin*. There are three states of *Ketsu*, namely, *Ketsu-Netsu (Ketsu-Jitsu) (*blood-heat), *Oketsu (Ketsu-Tai)* (blood stagnation) and *Ketsu-Kyo* (blood deficiency). Symptoms of blood-heat shows hematemesis, bloody stools, nose bleeds, for example and shows bleeding from tissue, constipation and yellowish urine [8]. When heated blood (*ketsu-netsu*) goes up to the upper body, it causes not only *oketsu* (blood stagnation) but also affects emotions such as irritation. Blood deficiency is the decay of the recuperation ability of blood. It causes so-called anaemia but also shows other dysfunction such as anorexia and weakening of digestion and absorption and these are governed by *Ki*. Blood will not circulate the body if *Ki* is insufficient to operate the blood vessels. This *Ki* in each part of the body is called *Ei-ki* or *Yin*-energy. *Ki* initiates functions of each organ of the body. *Ketsu* belongs to *Yin*. The concept of *Oketsu* or blood stagnation is the most important concept. *Oketsu* is a pathological change that would cause diseases when the blood is stagnated in the entire body or in a local tissue (Figure 5).

*Sansho* (triple heater), one of the 12 main acupuncture channels in the body, responsible for moving energy between the upper body and the lower body.

#### 2.5.3. Oketsu

**Figure 6:**
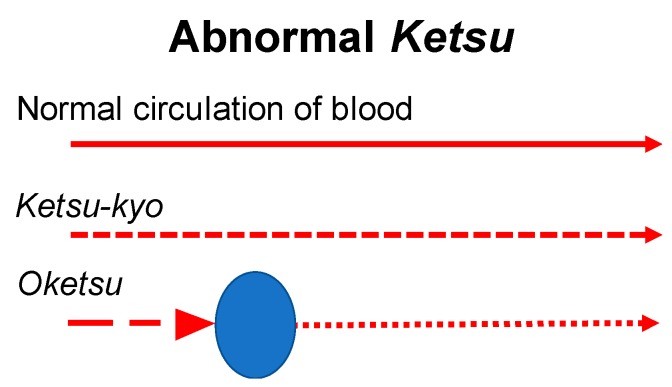
The Symptoms of *Oketsu*, in blood stagnation.

Pathological condition of *Oketsu* (blood stagnation) is expressed in a unique way to Kampo medicine and it is the most important concept [8]. In the modern medicine, it is perhaps more commonly understood as one of the syndromes of disability of microcirculation mechanism (Figure 6). However, Kampo medicine does not simply diagnose the symptoms but it considers important to identify the pathological condition which caused the symptom and the following are considered the pathogenic *Sho* of *Oketsu* such as the changes in the blood vessels due to inflammation, accentuation of blood coagulation factors, blood congestion, polycythaemia, menstruation, pregnancy or child-delivery. 

When the following symptoms are observed, we diagnose them *Oketsu*; the patient feels mouth dryness and would moisten the mouth with water but does not want to drink water; the patient feels stomach fullness though the abdominal distension is not observed; burning fever is felt locally or universally; purple spots appear on the skin or membrane; purple spots are appearing on the skin or membrane; dark purple spots appear on the edge of the tongue and lips are pale; stool is black; easy bleeding. The endogenous factor is in imbalance of autonomic nerve and the exogenous factor is coldness and bruise.

#### 2.5.4. *Sui, Aua*

**Figure 7:**
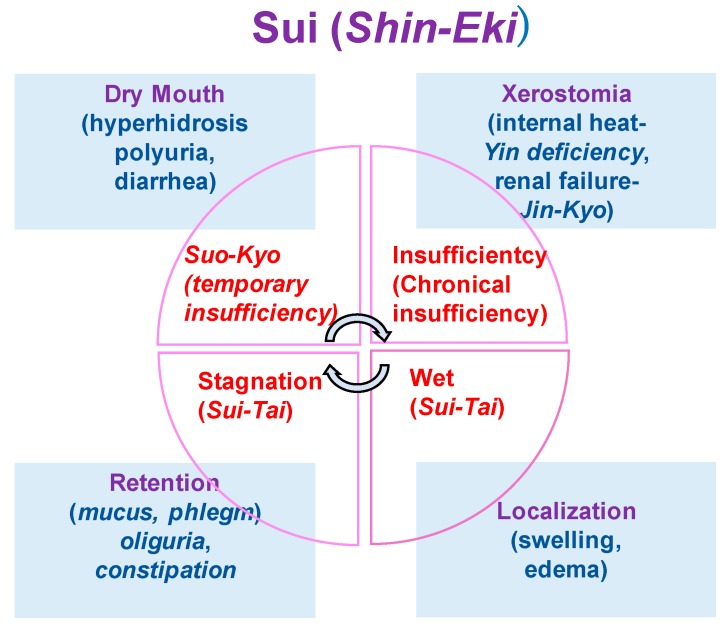
The concepts of *Sui* (*Shin-Eki*).

*Sui* is also called *Shin-Eki*. *Shin* is the relatively thin and pure fluids such as fluid component of blood, tissue fluids, sweat and urine, while *Eki* is relatively thick and sticky fluids among the intracellular and secretory fluids [8]. Each shows *Sho* of *Sui-Tai* (*Sui-Jitsu)* and *Sui-Kyo*. *Sui-tai* means stagnation of the body fluid (Figure 7).

Unevenly distributed *Sui* or aqua causes a local oedema. When it is linked with blood-heat, it becomes *Tan-In*-diseases and the fluid becomes sticky phlegm. As the blood-heat is understood as the inflammatory blood, it can appear when physical infection control is conducted. *Ko-Katsu* or mouth dryness is a symptom appearing when water is temporarily exhausted due to insufficient intake of water. Fever, thirst and tongue dryness are also systemically observed. It is called *Sui-Kyo* or aqua deficiency, which occurs from the temporal water exhaustion and shows dehydration. Xerostomia is a symptom of chronical insufficiency of water. Patients would feel mouth dryness and appeal lip dryness and cracks but would not want to drink water. It is mainly caused by the endogenous factors and systemically showing *Yin-Kyo* or *Yin*-deficiency and is diagnosed as *Oketsu-Sho.* It also shows the deterioration of kidney functions that is the symptom of deterioration of *Ki.*

#### 2.5.5. Views on Periodontal Disease and Toothache

**Figure 8:**
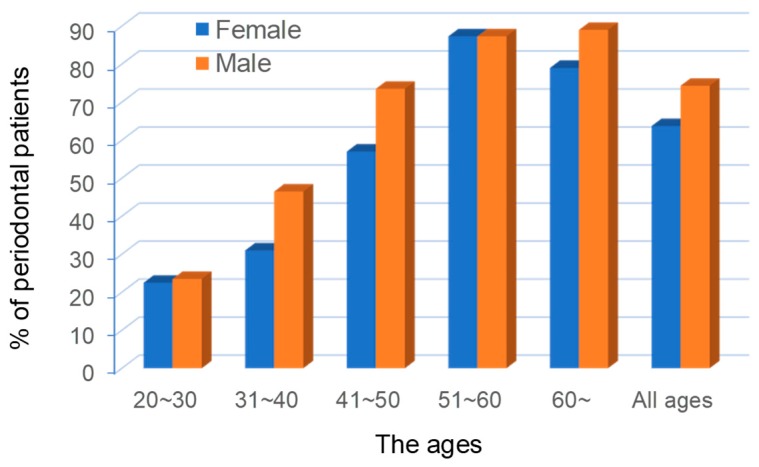
The proportions of poor blood circulation (*Oketu*) in the ages of the periodontal patients. Cited from [9] with permission.

In the progress of periodontitis, the gingival blood circulation induces the loss of capillary vessels and become anaemia. This status is so-called *Ketsu-Kyo* (blood deficiency) in Kampo. *Ketsu-Kyo* affects the nutritional disorder of gingiva and reduces oral immune system. Consequently, microcirculatory dysfunction induces the loss of oral biological activity, increasing the numbers of compromised hosts. This status is called *Oketsu* (blood stasis) in Kampo. *Oketsu* was observed in 70% to 80% of the randomly chosen periodontal patients above age 40 (Figure 8).

Although there are Kampo medicines which contain analgesic effect but they are not comparable to the western medicines. In oriental medicine, pain is understood as the change of symptom to maintain the homeostasis of organism. The flows of *ki*, *ketsu, sui* (vital energy, blood circulation function and aqua) deteriorate and it causes the unwell condition from stagnation. Therefore, Kampo medicine emphasizes to recuperate the body condition to normal. The background of pain would include not only infections and injuries but also the environmental and mental stress, where psychological factor would cause anxiety and anger and can go back to the emotions in the past. Chinese proverb says that good circulation brings no pain (= stagnation), meaning that if it flows, no pain.

**Figure 9:**
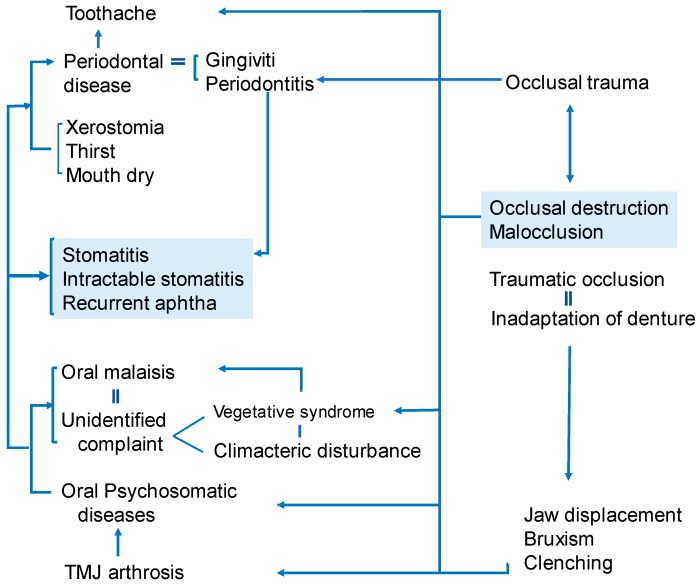
The correlations between occlusal-related troubles and oral diseases. (Watanabe S, unpublished data)

Occlusal trauma, occlusal destruction and traumatic occlusion are intraoral fragility and could cause systemic symptoms (Figure 9). 

## 3. Tongue Diagnosis

Clinical tongue conditions reflect the diseases of internal organs. The important points of clinical observations are colours, coatings and volume of the tongue [8]. The tension of the tongue (shape and lustre) change to the depending on the fall and rise of *Ki-Ketsu and* the location and the depth of the pathogen in body. Coating of the tongue mostly indicates the stomach condition, especially, dampness-dryness, coldness-heat of the body. Normal and abnormal conditions are shown in Table 4 and Table 5.

Kampo promotes the metabolism involved in the physical growth, development and physiological activity. Kampo moderate *Ketsu* and *Sui*, so that the blood runs in blood vessels and prevents blood from leaking outside. Kampo produces *Ki* that works to produce *Ketsu* and *Sui,* thus converting fluids into sweat and urine.

**Figure 10:**
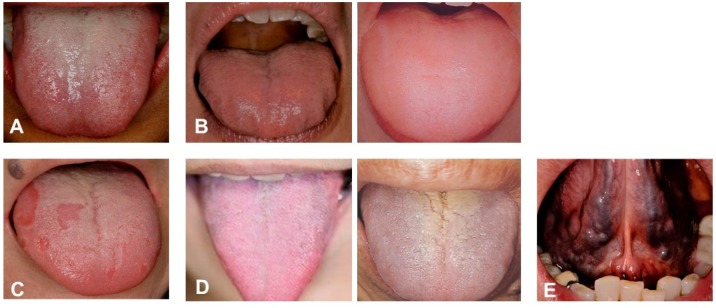
Tongue diagnosis of patients. (**A**) *Clinical observation of Healthy tongue*. Properly moistened: tongue coating white colour, thinly spread across the tongue. Light pink colour, Shape: fitting within the mouth. Back of tongue: sublingual vein is not over swelling. (**B**) *Abnormal tongue (Sui)*. Tongue peripheral impression, swelling tongue (oedema). Left: *Sui-Tai* or water stagnationis oedema, tongue peripheral impression. Pressure impression by the teeth on the edge of the tongue where water is sustained. Right: *Sui*-*Kyo* (insufficient water): oedema, water is not flowing well; big and swollen. (**C**) *Abnormal Ki (vital energy)*. Map-like tongue, insufficient Ki, slow circulation of *Ki*. Warming: Workings of warm-up and circulation; central of *Ki*. Defence: Workings to promote natural healing; protects skin and membrane and defends the body from the cause of disease. (**D**) *Abnormal tongue (Ketsu-Kyo and Kyo-Netsu)*. *Ketsu* (blood circulation) indicate the body of the tongue and sublingual vein that are in reddish purple, colour change of the tongue coating (*Ketsu*-*Kyo*, *Kyo*-*Netsu*). Left: *Ketsu*-*Kyo*: Tongue is thin and lean; Nutrition, water and blood circulation were insufficient. Right: *Kyo*-*Netsu*: Sticky blood; stagnated; Nutrition. water and blood body fluids were insufficient. Body is dry, since the heat remains to circulate internally. (**E**) *Abnormal tongue (Okesu)*. Blood heat condition in tongue. Ischemia (tongue: cyanosis) and over-swelling of sublingual veins. Systemically impaired flow of blood. These photos were taken, after obtaining the informed consent from the patients, under the condition that the patients are not identified. (Watanabe S, unpublished data)

Changes in the internal organs (liver, heart, lienogastric, lung and kidney) are reflected on the tongue (Figure 10). It tells the *Sho* of disease, namely, *Kyo, Jitsu, Hyo* and *Ri*. Tongue diagnosis is very important among the empirical diagnostic techniques. Systematic diagnosis theory has been formed based on the empirical evidences over several thousands of years. The rise and fall of *Ki, Ketsu* and *Sui* reflect the advance or retreat of illness. Severity of the disease can be seen on the tongue. Therefore, pathological changes of a disease are shown in the change of the tongue at its early stage. It is called *Mibyo* or pre-symptomatic in Kampo medicine. It is important diagnostic criteria and is recognized as a clue to find the cause of the illness at an early stage in the primary care.

## 4. Therapeutic Effect of *Juzentaihoto*

Periodontitis is the most common chronic inflammatory disease in humans and is characterized by alveolar bone loss and connective tissue destruction. Periodontitis is exacerbated by risk factors including age, gender, smoking, systematic diseases and psychological stress [10]. The stress response mediates the interaction between unfavourable psychological conditions and inflammatory periodontal disease. There is a higher prevalence of chronic destructive periodontal disease in individuals with psychological stress, which may be associated with acute necrotizing periodontal disease [11]. Psychological stress downregulates the cellular immune response, which may link the stress to periodontal disease [12]. Herbal medicines, such as *Juzentaihoto* (JTT), have good therapeutic effects for stress-related systematic diseases and minimal side effects. JTT consists of 10 herbs: *Ginseng radix*, *Astragali radix*, *Angelicae radix*, *Rehmanniae radix*, *Atractylodis lanceae rhizoma*, *Cinnamomi cortex*, *Poria*, *Paeoniae radix*, *Ligustici rhizome* and *Glycyrrhizae radix*. JTT is traditionally used for anaemia, rheumatoid arthritis, chronic fatigue syndrome and inflammatory bowel disease. It is widely used to prevent cancer metastasis and infection in immunocompromised patients [13]. We had examined the efficacy of JTT to prevent periodontitis.

### 4.1. Biological Activity (in Vitro) 

#### 4.1.1. Bactericidal Effect of JTT on *P. gingivalis*

**Figure 11:**
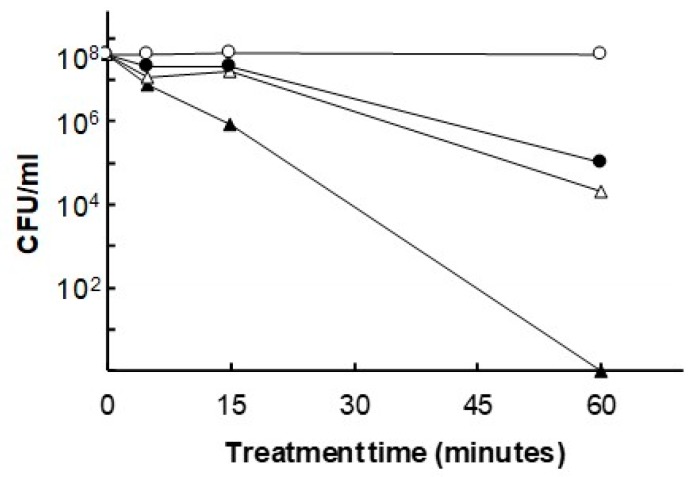
Antibacterial effect of JTT on *P. gingivalis*. Bacterial cells were treated with 10 mg/mL (▲), 1 mg/mL (△) or 0.1 mg/mL of JTT (●) or 0 mg/mL of JTT (〇) for the indicated period. At the end of the incubation period, a 10-fold serial dilution was performed in phosphate-buffered saline (PBS; pH 7.4) and spread onto a BHI blood agar plate broth supplemented with hemin (5 µg/mL), vitamin K_1_ (0.2 µg/mL) and yeast extract (5 mg/mL). The number of CFU (colony forming unit) was determined after 7 days of incubation under anaerobic conditions (CO_2_: 10%, H_2_: 10%, N_2_: 80%) at 37°C. Cited from [14] with permission.

We evaluated the bactericidal effect of JTT on *P. gingivalis*. Treatment of *P. gingivalis* with 0.1 to 10 mg/mL JTT reduced the number of viable cells in a dose-dependent manner. In particular, bacterial reduction by 10 mg/mL JTT was greater than that of 0 (control), 0.1 and 1 mg/mL JTX treatment at 60 minutes [14] (Figure 11), suggesting that JTT may suppress virulence factors from periodontal bacteria and prevent the progression of periodontitis. Some herbs from JTT and their ingredients have antimicrobial effects [15]. Compounds from *Glycyrrhizae radix* inhibit the growth of *P. gingivalis* [16]. *Glycyrrhizae radix* may have an important role in the bactericidal effect of JTT on *P. gingivalis*.

#### 4.1.2. Anti-osteoclastogenesis Effect of JTT

**Figure 12:**
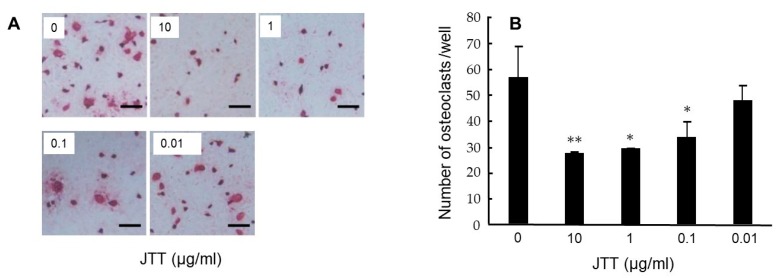
JTT inhibits osteoclast differentiation of BALB/c mouse bone marrow cells co-cultured with MC3T3-G2/PA6 cells. After incubation for 7 days, co-cultured cells were stained for TRAP (**A**) and determination of TRAP-positive multinucleated cells containing three or more nuclei (**B**). Results are expressed as the mean ±SD of triplicate cultures. **p < 0.01, *p < 0.05. Cited from [14] with permission.

We investigated whether JTT inhibits the osteoclast differentiation using a mouse co-culture system, according to the guideline of the intramural Committee of Ethics on Animal Experiments. Bone marrow cells (1.5×10^5^ cells/well) obtained from the tibiae of 5–8-week-old BALB/c mice and pre-adipose cell line MC3T3-G2/PA6 cells (1.5×10^4^ cells/well) were co-cultured for 7 days in the presence of 10 nM 1a,25-(OH)_2_D_3_ (calcitriol) and 10 nM dexamethasone in α modification of Minimum Essential Medium (α-MEM) supplemented with 20% FBS (foetal bovine serum) in 48-well plates under a 5% CO_2_ atmosphere. Osteoclast was defined as tartrate-resistant acid phosphatase (TRAP)-positive multinucleated cells containing three or more nuclei. Treatment with JTT at concentrations of 10, 1 and 0.1 μg/mL significantly inhibited osteoclast formation (Figure 12A) [14]. A low concentration (0.1 µg/mL) of JTX significantly inhibited the osteoclast formation compared to control (Figure 12B). *Angelicae gigantis radix*, one of the components in JTT, significantly decreased osteoclast formation [17]. Therefore, JTT can be a therapeutic drug that prevents periodontitis. 

### 4.2. Clinical of JTT 

**Figure 13:**
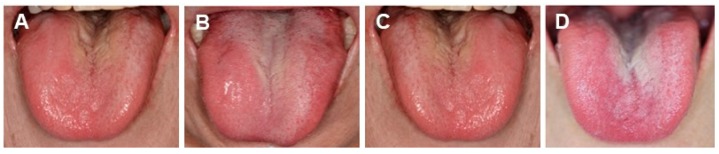
Changes of the tongue surfaces at 1 (**A**), 4 (**B**), 8 (**C**) or 12 (**D**) months after GRS + JTT treatment. (Presented in Kanagawa Dental College Society 53rd General Assembly: A case in which Kampo and denture adjustment was successful for patients complaining of denture). Photos were taken, after obtaining the informed consent from the patient. (Watanabe S, unpublished data)

Kampo medicine and adjustment of denture were effective for a patient (80 years old, female, cervical cancer operated 25 years ago with good prognosis), with symptom of spinal canal stenosis, cervical vertebra stenosis, dizziness, unsteadiness and oppression on the chest. She was diagnosed as inadaptation of the denture, oral malaise (tongue), dysfunction of masticatory and xerostomia. Her *oketsu* (blood stagnation) was improved by taking JTT mornings and evenings – 2 doses before meals. Swelling and oedema of the tongue was improved by taking *Goreisan* (GRS) before going to bed. Medical consultation resulted in the improvement of mental condition. Two Kampo medicines, GRS (that improves *Sui*-*Tai* or fluid retention symptom caused by poor metabolism of water) and JTT [that enhances *ki* (vital energy) and *ketsu* (blood circulation function) and improve fatigue, anaemia, low appetite, night sweat, cold hands and feet which accompany the decondition] were administered in this case. In the initial diagnosis, prescription of GRS showed no progress in 2 weeks, then additionally JTT *was* administered. After 1 month later (Figure 13A), the filling pain when chewing with denture stopped jaws gliding and oppression on throat while sleeping. Two months later, she could bite off and eat food. She had no sensation of tongue torsion, stopped waking up at night due to the neck pain. Administration of GRS stopped due to frequent urination. Four months later (Figure 13B), upon the mounting of dentures, she felt neck strain and torsion of denture and tongue. The administration of GRS was restarted. Eight months later (Figure 13C), she became able to chew any food, with no sensation of tongue swells. She did not wake up at night. Twelve months later (Figure 13D), the administration of GRS was stopped but that of JTT was continued (Figure 13).

When making dental prosthesis, it is necessary to pay attention to the intraoral environment. In the case above, we observed the lower jaw denture floated due to tongue oedema and swelling and that the denture instability caused the occlusal pain and dysfunction of masticatory. We therefore prescribed Kampo medicines that would improve these symptoms. In this clinical case, we considered that the improvement of *sui-tai* (fluid stagnation) should be the target of *Hyo-Chi*, a local and symptomatic treatment, to improve the tongue oedema, so GRS was administered. However, we could not get the expected results, so JTT was also administered in the morning and before going to bed and it presented the trend of improvements from the next day. JTT is a Kampo medicine for *hon-chi*, that is, the systemic treatment of the fundamental cause of the disease. It is often observed in the dentistry and intraoral medicine that patient’s conditions change until the symptom finally surfaces due to the chronical deficiencies. Especially, the entire body is psychologically and mentally affected when malocclusion exists. Under the situation that malocclusion lasts long, it is known that glucocorticoid appears in blood chronically due to the chronical stress from the malocclusion which would induce the malfunction of negative feedback. The Oriental medicine explains that the symptoms of *Ki* and *Ketsu* occur and they worsen as the time elapses. In our case above, GRS manifested its effect at early timing by the simultaneous administration of JTT. It not only cured the oedema of the tongue but also mentally stabilized the patient. 

JTT is known to improve the pathological condition of the blood circulation and mental stability. When the condition of the disease is found difficult to improve, Kampo medicine of *hon-chi* (treatment of fundamental cause), in combination would relieve the symptoms. 

## 5. Therapeutic Effects of *Jixueteng*

### 5.1. Bactericidal Effect of Jixueteng

**Figure 14:**
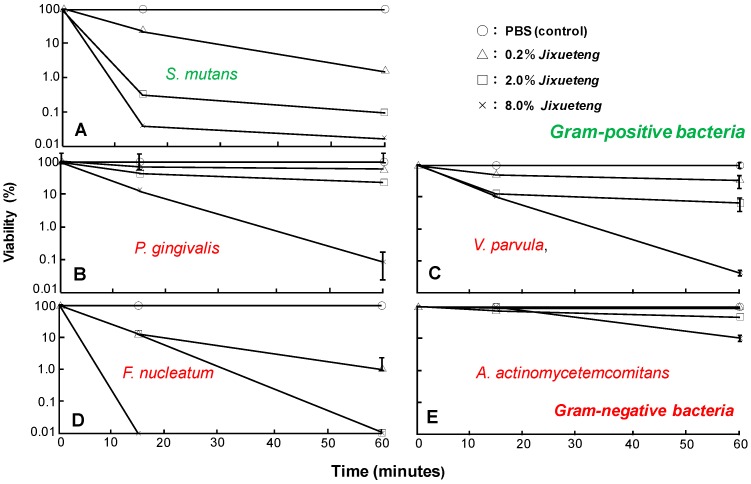
Bactericidal effect of *Jixueteng* against Gram-positive and -negative bacteria. Gram-positive bacteria (*S. mutans)* (**A**) and gram-negative bacteria (**B**: *P. gingivalis*, **C**: *V. parvula*, **D**: *F. nucleatum*, **E**: *A. actinomycetemcomitans*.) were treated by 0.2, 2 and 8% of the *Jixueteng* extract for 1, 15 or 60 min. The suspensions were treated by PBS as a control. Cell viability was expressed as a percentage relative to control. Cited from [23] with permission.

*Jixueteng* is prepared from the dried stems of *Spatholobus suberectus* (*S. suberectus*) Dunn of the family Leguminosae. *Jixueteng* has beneficial pharmacological properties such as increasing circulation, analgesia and the number of red and white blood cells [18]. *Jixueteng* contains various types of flavonoids such as flavone, isoflavones, flavanones, flavanonols and chalcone [19]. Flavonoids are natural products that show antibacterial [20] and antioxidant activities [21]. Production of reactive oxygen species (ROS) is decreased by *Jixueteng* in a dose-dependent manner [22]. Therefore, we focused on the bactericidal effect of *Jixueteng* on oral bacteria. The gram-positive species, *Streptococcus mutans* Ingbritt (*S. mutans*) and the gram-negative species, *Aggregatibacter actinomycetemcomitans* ATCC 29523 (*A. actinomycetemcomitans*), *Fusobacterium nucleatum* ATCC 25586 (*F. nucleatum*), *Porphyromonas gingivalis* ATCC 33277 (*P. gingivalis*) and *Veillonella parvula* GAI-0580 (*V. parvula*), were grown in BHI broth and suspended in PBS to an optical density of 1.0 at 600 nm. Fifty µL of bacterial suspension was exposed for 1, 15 and 60 min in the presence of 0, 0.2, 2.0 or 8% *Jixueteng* extract. The same volume of PBS was used as a control. At the end of the incubation period, a 10-fold serial dilution was inoculated onto BHI sheep blood agar plates and incubated anaerobically at 37°C for 7 days. The bactericidal effect of *Jixueteng* was determined by counting the number of bacterial cells. *Jixueteng* extract reduced the number of viable bacterial cells, such as *S. mutans* (Figure 14A), *P. gingivalis* (Figure 14B), *V. parvula* (Figure 14C) and *F. nucleatum (*Figure 14D*)* (Figure 14). In particular, the bactericidal effects of *Jixueteng* against *F. nucleatum* (Figure 14D) was higher than those of other oral bacteria. After treatment with 8% *Jixueteng* extract for 60 min, the number of *P. gingivalis* was decreased from 4.61 × 10^9^ to 2.90 × 10^6^ per millilitre (Figure 14B) [23]. Gram-negative periodontal pathogens are late colonizers of dental plaque and promote inflammatory tissue destruction in the oral cavity [24]. Thus, *Jixueteng* extract may act selectively on periodontal bacteria and break down dental plaque accumulation.

### 5.2. Inhibitory Effect of Jixueteng on Osteoblast Differentiation

In periodontitis, several cytokines, such as interleukin (IL)-1, prostaglandin (PG) E_2_ and RANKL (receptor activator of NF-κB ligand), promote osteoclast differentiation. RANKL, a tumour necrosis factor (TNF)-family member, binds to its receptor RANK, which is on the surface of osteoclasts and preosteoclasts. The interaction between RANK and RANKL signalling is important for osteoclastogenesis [25]. To examine the influence of *Jixueteng* on osteoclastogenesis, we used mouse co-cultured cells in the presence of 1α,25-(OH)_2_D_3_ and dexamethasone. *Jixueteng* extracts were added to co-cultured cells at a final concentration of 0.1%, 0.01%, 0.001% and 0.0001% and cultivated for 7 days under 5% CO_2_ atmosphere. After 7 days, cells were fixed and stained for TRAP. TRAP-positive multinucleated cells containing three of more nuclei were counted as osteoclasts. The treatment of *Jixueteng* extract (at concentrations of 0.1% and 0.01%) significantly inhibited osteoclast formation (P < 0.01). Addition of 0.1% extract completely inhibited TRAP-positive cells and multinucleated osteoclasts. In addition, the inhibitory effect of *Jixueteng* on osteoclast survival was determined by mouse co-cultured cells in the presence of RANKL and PGE_2_. The number of osteoclasts was decreased with 0.001 to 0.1 mg/mL *Jixueteng* in a dose-dependent manner [26]. These results suggest that *Jixueteng* inhibits osteoclastogenesis and reduces osteoclast activity in periodontitis.

### 5.3. Inhibitiory Effect of Alveolar Bone Resorption by Jixueteng on Mice Experimental Periodontitis

**Figure 15:**
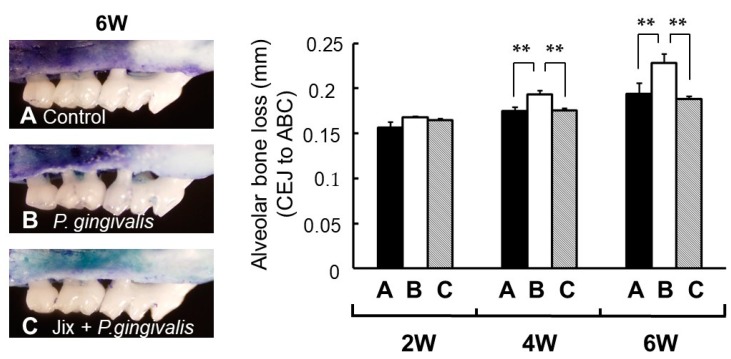
Morphometric bone levels of 6 week after *P. gingivalis* infection (left) and alveolar bone levels at 2, 4 and 6 weeks after *P. gingivalis* infection (right). **A**, non-infected control; **B**, infected with *P. gingivalis*; **C**, Jixueteng administered group along with *P. gingivalis* infection. Bone levels were evaluated by measuring the distance from the cemento-enamel junction (CEJ) to the alveolar bone crest (ABC) at seven palatal sites per mouse. Values indicate the mean bone loss levels ± standard error of the mean (n = 6/group). **: significantly different (*p* < 0.01). Cited from [28] with permission.

Flavonoids are effective ingredients for the inhibition of inflammatory bone resorption [27]. We evaluated the inhibitory effect of alveolar bone resorption by *Jixueteng* using an experimental periodontitis model, under the guideline of the intramural Committee of Ethics on Animal Experiments. Fifty-four male C57BL/6N 4-week-old mice were used. Mice were given sulfamethoxazole (1 mg/mL) and trimethoprim (200 mg/mL) in their drinking water for 4 days to reduce original oral flora followed by 3 days of an antibiotic-free period before bacterial infection. The bacteria used was *P. gingivalis* A, which was inoculated in BHI broth under anaerobic conditions. Animals were randomly divided into the following three groups: Group A received only 5% carboxymethylcellulose (CMC) (sham-infected group), group B was infected orally with *P. gingivalis* and group C was administered *Jixueteng* extract in drinking water and was infected orally with *P. gingivalis.* Each mouse in group B and group C was infected orally with *P. gingivalis*, which was suspended in 5% CMC and received 0.1 ml (1.0 × 10^10^ cells/mL) of bacterial suspension. The bacterial infection was given by oral gavage (three times) at 48 h intervals. The mice were sacrificed 2, 4 and 6 weeks after the final bacterial infection to examine the change in alveolar bone resorption every 2 weeks. The left sides of the horizontal alveolar bone resorption around the maxillary molars were evaluated morphometrically as dry specimens to measure horizontal alveolar bone loss. The distance between the cemento-enamel junction (CEJ) and the alveolar bone crest (ABC) was measured at seven palatal sites per mouse. Measurements were made under a dissecting microscope (40× magnification) fitted with a digital high-definition system, standardized to provide measurements in millimetres. The right sides of the upper jaws were analysed for histology. The samples were fixed, decalcified and embedded in paraffin. The paraffin section was cut serially into 5-mm sections in a mesial–distal direction. The sections were stained for haematoxylin–eosin (H–E) and TRAP. In particular, TRAP-positive multinucleated cells were defined as osteoclasts and examined under an optical microscope (40× magnification). The number of osteoclasts was counted in the area of the periodontal tissue between the mesial root of the first molar and the distal root of the third molars. We found apparent horizontal bone loss in C57BL/6N mice challenged with *P. gingivalis* (group B) but not in the control (group A) or the *Jixueteng*-administered group (group C) (Figure 15A). Figure 15B shows the mean values ± standard error (SEM) of the CEJ to ABC derived from seven measurement sites in weeks 2, 4 and 6 after infection. Induction of alveolar bone loss was more reproducible with an infection by *P. gingivalis* by oral gavage (group B) (*p* < 0.01) than in the sham-infected control (group A) 4 weeks after infection, whereas no difference was observed 2 weeks after infection. In all experimental groups, the maximum resorption of alveolar bone was observed at the end of the experiment and the mean bone levels of the sham-infected control (group A) and *P. gingivalis* infection group (group B) were 0.194 ± 0.001 mm and 0.228 ± 0.010 mm, respectively. Alveolar bone loss was significantly lower in the *Jixueteng* group (group C) (*p* < 0.01) than that of group B in weeks 4 and 6. The mean bone level of group C in week 6 was 0.188 ± 0.003 mm, which was comparable to that of the control group A (Figure 15) [28]. 

**Figure 16:**
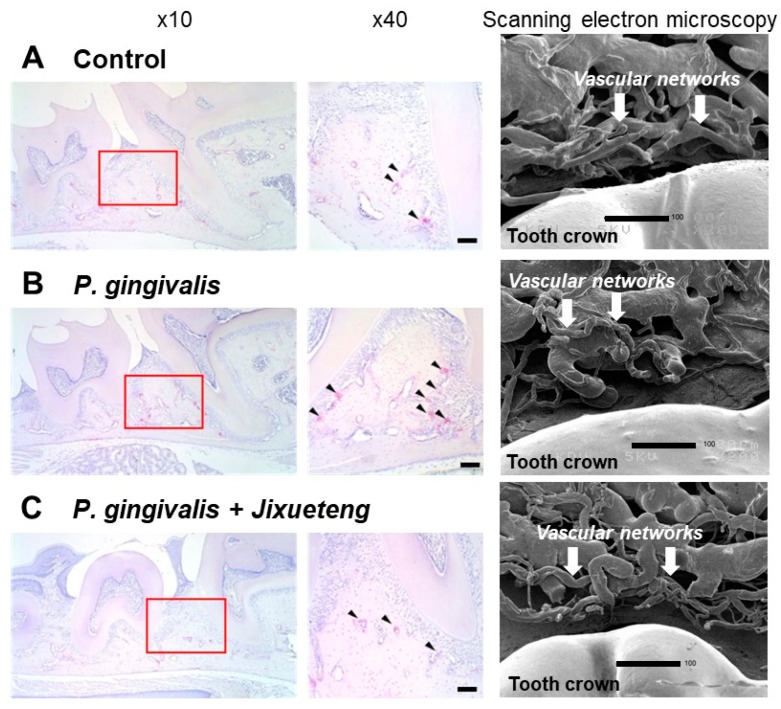
Histopathological examination of mice periodontal tissues. Specimens obtained from the maxillary bone of mice were evaluated with TRAP staining. Osteoclasts (arrows) were observed along the alveolar septum of the maxillary molars. **A**, con-infected control; **B**, infected with *P. gingivalis*; **C**, administered *Jixueteng* and infected with *P. gingivalis*. Original magnification: × 10 and × 40. Bars: 100 μm. Scanning electron microscopy shows that compared to the normal group (**A**), morphological degeneration of vessels in vascular networks and abnormality of the vascular lumen caused by *P. gingivalis* infection were observed (**B**). However, improvement in degeneration of these vascular networks and prolongation of the vascular plexus were observed by administration of *Jixueteng* (**C**). Cited from [28] with permission.

By histopathological examination, osteoclasts were observed along the alveolar septum in mice periodontal tissues (Figure 16). Table 6 shows the number of osteoclasts in the alveolar bone crest. No significant difference in the number of osteoclasts was observed among the experimental groups 2 weeks after infection.

After 4 weeks, the number of osteoclasts in the *P. gingivalis*-infected group B was significantly higher than that of sham-infected group (group A) and the *Jixueteng*-administered group (group C) (*p* < 0.01) [28]. *P. gingivalis* has virulent factors that induce inflammatory responses and alveolar bone resorption [29]. This bacterium also invades and survives in host cells, inducing a network of inflammatory responses [30]. *P. gingivalis* also increases the likelihood of systemic diseases such as diabetes and cardiovascular disease [31]. We previously reported that *Jixueteng* improves gingival vascular networks in a *P. gingivalis*-induced periodontitis [28] (Figure 15). *Jixueteng* may inhibit the adherence and colonization of *P. gingivalis* in mice oral cavities. Therefore, our findings suggest that *Jixueteng* reduces the inflammatory destruction in periodontitis. *Jixueteng* has bactericidal effects against oral bacteria, inhibits the osteoclastogenesis and reduces the alveolar bone resorption induced by *P. gingivalis*. *Jixueteng* reduces the inflammatory tissue destruction in periodontitis and *Jixueteng* may be a useful ingredient to prevent periodontitis.

## 6. Therapeutic Effects of *Mastic*

### 6.1. History of Mastic (Kampo Name: Yo-Nyuko olibanum)

**Figure 17:**
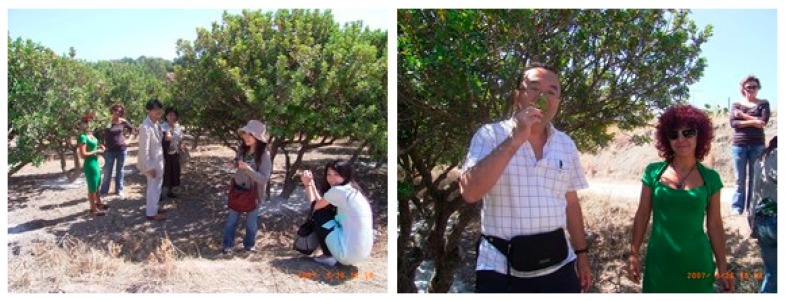
Mastic tree (*Pistacia lentiscus*). (photos taken at Chios island, Greece, 2007)

Mastic is the resin collected from the naturally growing trees in only Chios Island in southeast Aegean Sea of Greece. It was initially called frankincense. Mastic has a long history, it is written in the Old Testament (Genesis 37:25) and many ancient Greek literatures mentioned the medical effect of mastic. Christopher Columbus, before he stayed in Portugal, visited Chios Island during his voyage to the Orient as recorded in his diary in 1474–1475 and he described about mastic; it is sticky sap extracted from tree and becomes resin when solidified. There has been a habit of chewing this in Greece since more than 5000 years ago and it was known that those people who had this habit rarely had digestive diseases. In China’s classical Kampo medicine masterpiece of “Zu-Kei Honzo” (masterpiece of plant diagrams) also described it as *Kun-roku-ko*, kuduruka. The substance called frankincense today is the resin from the trees of *Boswellia* genus of the *Burseraceae* family that grow in north-eastern Africa or Arabian coast and it is different from the mastic from Greece. Traditional frankincense to present is the same kuduruka in the book of Honzo and they grow in the Mediterranean coast areas. Resin from the tree that belongs to the *Anacardiaceae* family is considered *MASTICHE RESINA*. This is called “mastic” or *Yo-Nyuko* in Kampo and is known as *Pistacia lentiscus* locally (Figure 17).

It is empirically proven that herbs that are used in folk therapies, including the herbal medicines constituting Kampo, have multifunctional medicinal effects. Mastic resin has a unique shape and various efficacy and has been used to promote health from old time. In addition, mastic resin has been used in chewing gums as a material for oral health and hygiene and indicated antiplaque activities. Recently in Japan, mastic has been receiving attention as material for oral cares and many companies are developing and selling mastic-formulated oral gels for toothbrushing paste. 

Mastic has been forming a market of high-end products of oral cares. Also, dentists have been paying attentions to its effect and mastic is securing its position in the clinical medicine as the primary care product for oral cavity cares. To cope with the improvements of adult diseases and systemic diseases associated with the oral hygiene in the aging society of Japan and further to improve the oral health in Asia, a group of dentists launched an NPO called “Mastic Clinical Study Group.” It has been running the public awareness building programs of the oral hygiene including preventive dentistry. Presently, many dentists are making use of mastic in the clinical studies and are promoting its use in the treatment of patients and to spread the awareness of the primary cares.

### 6.2. Component of Mastic 

Essential oils, obtained by hydrodistillation of aerial parts of *Pistacia lentiscus* var *chia*, were determined for their oil composition using gas chromatography-mass spectrometry (GC/MS). Most abundant component was α-pinene (72.93% of the total oil composition), followed by β-myrcene (13.57%) > β-pinene (2.58%) > limonene (0.89%) > linalool (0.73%) > camphene (0.58%), methylanisol (0.58%) > α-pinene oxide (0.56%) > sabinene (0.30%), β-caryophyllene (0.30%) > verbenone (0.26%) > pinocarveol (0.21%) > myrtenol (0.18%) > pinocarvone (0.10%) [32]. 

### 6.3. Biological Activity of Mastic Gum (Resin) 

#### 6.3.1. Antimicrobial Activity

**Figure 18:**
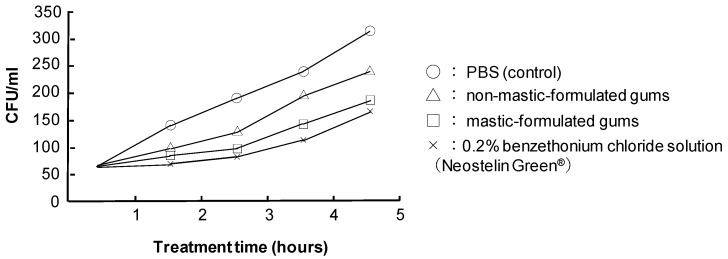
Bactericidal effect of mastic gum against oral bacteria. (Hamada N, unpublished data)

Compared with the group, which used the PBS mouthwash, the group that used the mastic-formulated gums showed the significant inhibition of the increase of oral bacteria (Figure 18). Compared with the group that used the gums without mastic formulation, it inhibited the increase of the pathogenic bacteria and the effect was equivalent of benzalkonium chloride. 

Stick examined the antibacterial effect against gram-negative bacteria. The result of the examination of the minimum inhibitory concentration (MIC) of mastic resin oil against the oral bacterial groups showed it had antibacterial effect (< 0.05%) for adult periodontal bacteria, *P. gingivalis*. Mastic rein oil also showed great selective effect at < 0.05% against *Fusobacterium nucleatum* (Table 7 and Table 8). *F. nucleatum* is an important periodontal bacterium that derives the bacterial agglutination on the dental plaque formation. Mastic rein oil can reduce the dental plaque and promote the prevention of periodontitis.

Mastic showed selective antimicrobial activity against *Porphyromonas gingivalis* and *Prevotella melaninogenica*, as compared with that against the growth of *Actinomyces viscosus*, *Streptococcus gordonii*, *Streptococcus mutans*, *Capnocytophaga ochracea*, *Fusobacterium nucleatum*, *Prevotella intermedia*, *Staphylococcus aureus*, *Escherichia coli* and *Candida albicans* [33]. When mastic gum was fractionated with successive extractions with organic solvents with increasing water-solubility into hexane, ethyl acetate, n-butanol extracts and remaining water layer, the ethyl acetate extractable fraction showed eight or nine times higher anti-bacterial activity against *Streptococcus mutans* (IC_50_ = 104 μg/mL), as compared with other fractions (831–936 μg/mL). The most sensitive bacterium was *P. gingivalis* (IC_50_ = 32.7 μg/mL), followed by *S. mutans* (IC_50_ = 104 μg/mL), *S. aureus* (IC_50_ = 609.4 μg/mL), *F. nucleatum* (IC_50_ = 759.6 μg/mL) and *E. coli* (IC_50_ = 907.4 μg/mL) [34]. Ethyl acetate extract of mastic, which has higher antibacterial activity than unfractionated mastic, may be appropriate for the treatment of periodontal diseases.

The MIC of polymers from mastic gum of *Pistacia lentiscose* (MW: 50-130 kD), isolated by gel permeation chromatography against gram-negative bacteria (*Escherichia coli* type 1, *Salmonella typhimurium*, *Serratia marscens*. *Pseudomonas aeruginosa*, *Alcaligenes faecalis*, *Enterobacter aerogenes*, *Pseudomonas fluorescens*, *Proteus vulgaris*, *Porphyromonas. gingivalis*) and gram-positive bacteria (*Bacillus cereus*, *Staphylococcus aureus*, *Streptococcus faecalis*, *Staphylococcus epidermidis*, *Bacillus subtilis*, *Corynebacterium sp*) was 200–250 and 1000 μg/mL, respectively. The MIC of polymer of β-myrcene (MW 50–500 kD), synthesized by incubation with cyclohexane and *sec*-butyl lithium, against gram-negative and -positive bacteria was 100 and 1000 μg/mL, respectively [35]. Chewing mastic gum decreased the total viable bacteria, *S. mutans* and *lactobacilli* in saliva in orthodontically treated patients with fixed appliances, suggesting the usefulness of chewing mastic gum in preventing caries lesions [36].

#### 6.3.2. Antiviral Activity

Anti-HIV activity was determined by the selectivity index (SI), based on the ratio of 50% cytotoxic concentration (CC_50_) against mock-infected CD4-positive human T-cell line MT-4 cells to 50% protective concentration (EC_50_) against HIV-infected MT-4 cells. All mastic extracts did not prevent HIV-induced cytopathic effects on MT-4 cells (SI <1), whereas three anti-HIV agents (azidothymidine, dideoxycytidine, curdlan sulphate) showed excellent anti-HIV activity (SI = 5624, 3868, 7142). All mastic extracts partially but significantly reduced the HSV-induced cytopathic effects on Vero cells, recovering the cell viability up to 43.2 ± 5.3% of mock-infected cells [34]. 

#### 6.3.3. Anti-tumour Activity

Mastic showed very low antitumor activity against four human oral squamous cell carcinoma cell lines (Ca9-22, HSC-2, HSC-3, HSC-4) (CC_50_ = 13.5–24.4 μg/mL) as compared with three human normal oral cells (gingival fibroblast HGF, periodontal ligament fibroblast HPLF, pulp cells HPC) (CC_50_ = 28.1–84.8 μg/mL), with the tumour-specificity value (TS, determined by the ratio of CC_50_ against normal cells to CC_50_ against tumour cells) of only 1.4 to 2.4. Among 5 extracts, ethyl acetate extract showed the highest TS values (TS = 2.6), although its values were two-order lower than that of doxorubicin (TS = 244.7) [34]. However, mastic showed approximately 5-fold higher cytotoxicity against human leukemic cell lines: promyelocytic HL-60 (19 μg/mL), myeloblastic ML-1 (25 μg/mL ), myeloblastic KG-1 (27 μg/mL), erythroleukemia K-562 (30 μg/mL), as compared with normal cells (HGF, HPLF, HPC) (93–155 μg/mL). Mastic induced apoptotic cell death (internucleosomal DNA fragmentation, caspase-3 activation, decline in the intracellular concentration of putrescine) in HL-60 promyelocytic leukaemia, while it inhibited the spontaneous apoptosis of oral polymorphonuclear leukocytes. Mastic showed hydroxyl radical scavenging activity, suggesting the beneficial effects of mastic on oral health [33]. Mastic gum (200 mg/kg) inhibited the growth of colorectal tumour xenografts by approximately 35%, when the optimal experimental conditions were chosen [37]. 

Recently, apoptosis induction by mastic in COLO205 human colonic adenocarcinoma [38], MCF-7 human breast cancer cells [39], LT97 human colon adenoma cells [40], FTC-133 (human follicular thyroid carcinoma) [41], H-SY5Y, SK-N-BE(2)C human neuroblastoma [42] and YD-10B human oral squamous carcinoma [43] has been reported, however, most of these studies have not mentioned the tumour-specificity of mastic. 

#### 6.3.4. Anti-inflammatory Activity

Essential oil of mastic showed a strong iron chelating activity (IC_50_ = 20 μg/mL) and actively scavenged hydroxyl radical (IC_50_ = 3 μg/mL) and protected *tert*-butyl hydroperoxide-treated lymphocyte [44]. Mastic inhibited the production of nitric oxide (NO) and prostaglandin (PG)E_2_, as well as expression of inducible NO synthase (iNOS) and cyclooxygenase (COX)-2 protein and mRNA, induced by lipopolysaccharide (LPS)-activated mouse macrophage-like RAW264.7 cells. Mastic scavenged hydroxyl radical more potently than NO and superoxide radicals. The narrow range of effective concentration of mastic due to its cytotoxicity may limit its potential application as an anti-inflammatory agent [45].

#### 6.3.5. Inhibition of CYPs

Five days oral treatment with *Pistacia lentiscus* oil 100 μL per mice did not show any undesirable effect on the function of kidney and liver but significantly inhibited the enzyme activity and expression of CYP1A1, CYP1A2, CYP2E1 and CYP3A4, especially in the liver tissue [46]. The result suggests the possibility that when mastic is used in combination with other pharmacological agents, the biological action of the latter may be more enhanced. 

Among five masic fractions, *n*-hexane extract exhibited the highest CYP3A4-inhibitory activity (IC_50_ = 3.1 μg/mL), followed by methanol extract (macerated) (IC_50_ = 4.1 μg/mL), *n*-butanol extract (IC_50_ = 12.1 μg/mL), unfractionated sample (IC_50_ = 14.3 μg/mL), ethyl acetate extract (IC_50_=14.8 μg/ml) (C) and, finally, methanol extract (refluxed) (IC_50_ = 24.4 μg/ml). Washing out these CYP3A4 inhibitory substance with *n*-hexane may reduce these pharmacological action or side-effects of combined drugs [34]. 

#### 6.3.6. Oral Application of Mastic Gel

**Figure 19:**
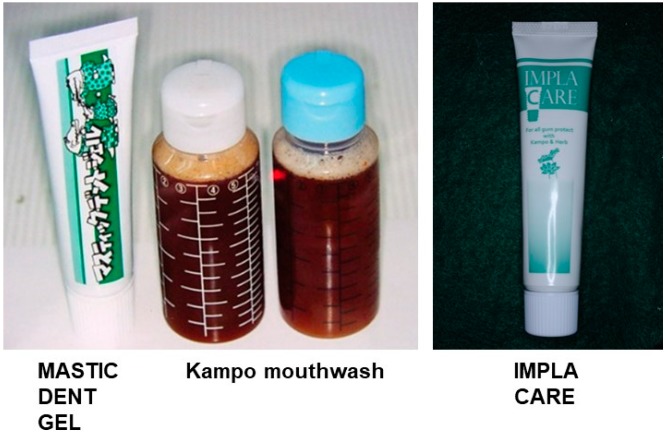
Toothpaste and mouth-rinse including mastic and IMPLA CARE. (unpublished data)

Salivary bacteria create healthy microbiomes when the intraoral condition becomes good. In order to maintain the good intraoral environment, we developed a gel toothpaste with mastic (*Boswellia carterii,* Kampo name: Yo-Nyuko), a mouthwash with Kampo herbs formulated (Figure 19). 

**Figure 20:**
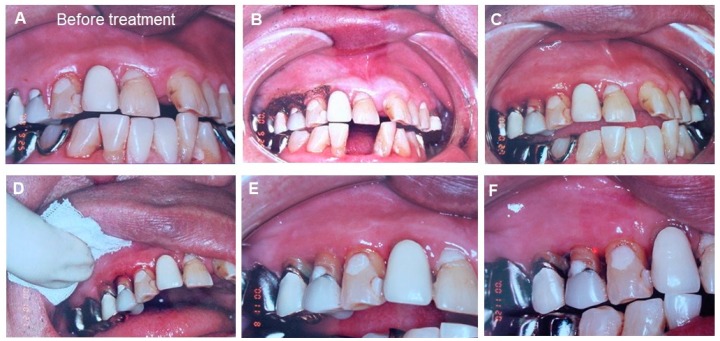
The clinical observation of periodontal tissue around the upper 4th and 5th teeth before and after treatment with laser and mastic gel. (**A**) Before treatment (at the first visited time); (**B**) First time after the laser treatment. Mastic gel was applied after carbonized laser treatment. (**C**) Second times after the laser treatment. After the laser treatment, chlorhexidine was used to prevent bacterial infection and mastic gel was applied at the gingiva. (**D**) Third times after the laser treatment. Mastic gel was applied after light coagulation layer was added by laser. (**E**) Fourth times after the laser treatment. Mastic gel was applied around the inflammatory gingival area. (**F**) Inflammatory gingival area between upper 4th and 5th are improved by using the mastic gel. Photos were taken, after obtaining the informed consent from the patient. (Watanabe S, unpublished data)

Since the patient had lost the freedom of his hands thus the prevention infection control after treatment would be difficult, it was decided to try treatments using dental laser and Kampo mouth wash and mastic gel to improve the gingival tissue [47,48,49]. Then, we show a clinical case using the mastic gel in oral cavity. The following is the clinical report of patients treated with mastic in our dental clinic, after obtaining the informed consent from the patient, under the condition that the patient is not identified (Figure 20). 

The patient was a 66 years old male. Showing bleeding due to the mobility of upper right 4th and 5th teeth. He visited us due to his chief complaint of mastication disorder. He had a stroke 3 years ago and is currently seeing the physician once a month. The condition is stable. As his hands tremble, the mouth cleaning was poor and the breath was bad. Poor oral hygiene, bleeding from the gums (+++), red swelling on the gums and inflammation were observed. As it was necessary to create the environment that treatment can be done, Kampo mouthwash and application of mastic gel toothpaste was conducted for three times a day before and after the treatment. Next, we showed the improvement of the chief complaint by the initial treatment. When the patient came to the clinic, his 4th and 5th teeth in upper right were showing medium level of mobility but it was judged from the observation using X-ray that the bone absorption was not bad.

We used the dentifrice that mastic was formulated and expected the effect of preventing the fixation of pathogen bacteria before and after the treatment. Considering that the oral hygiene significantly influences the management of the entire body, the biological study of the natural products is critically important from now on [50,51,52].

## 7. Development of A Dentifrice Gel Containing A Mastic Resin and *Jixueteng*

Periodontitis is the second most common dental disease worldwide after dental decay. Periodontitis is caused by microorganisms that adhere and grow on tooth surfaces and by an aggressive immune response against these microorganisms. The mouth contains a wide variety of oral bacteria, which is an ideal environment for their growth. Nutrition is supplied from food residues and saliva, nitrogen and amino acids mix in gingival crevicular fluids. Periodontitis is triggered by a complex microbial biofilm in the subgingival area that houses over 700 bacterial species and phylotypes [47]. Bacteria from the red complex group, such as *P. gingivalis*, predominate in gingivitis and periodontal disease patients by PCR analysis. Three microorganisms are mainly associated with periodontal disease, *Treponema denticola*, *Tannerella forsythia* and *P. gingivalis* and they usually form a complex called red complex bacteria (RCB). These bacteria are Gram negative, non-spore-forming anaerobic organisms and they may be found as pure or mixed infections [48]. RCB possess several virulence factors including fimbriae, proteinases, exopolysaccharides and hemin-binding proteins [49]. RCB have been detected in both subgingival plaque and in the apical root canal and cause periodontal and endodontic diseases [48,53,54]. *P. gingivalis* is a pathogen that causes periodontal disease, which is a common chronic inflammatory disease [55,56,57]. 

*Jixueteng* is a herbal medicine with pharmacological properties, such as increasing circulation, analgesia and the number of red and white blood cells and is composed of the dried stems of *Spatholobus suberectus* Dunn and *Millettia dielsiana* Harms, both family Leguminosae [58,59]. *Jixueteng* has potent local anti-infection effects on oral indigenous bacteria and inhibits alveolar bone loss [23,39]. These findings suggest that *Jixueteng* is a safe and effective therapeutic agent for periodontal disease because of its antibacterial and immune activities and its ability to improve circulation. However, *Jixueteng* has not been clinically used in the oral field and its effects on reactive oxygen species (ROS) in inflamed regions and the detailed mechanisms underlying these pharmacological actions remain unclear. ROS is involved in various physiological and pathological events. Overproduction of ROS causes oxidative damage to biomolecules, such as lipids, proteins and DNA, which ultimately results in many chronic diseases in humans such as atherosclerosis, cancer, diabetes, rheumatoid arthritis, post-ischemic perfusion injury, myocardial infarction, cardiovascular diseases, chronic inflammation, stroke, septic shock, aging and other degenerative diseases [60,61].

Because, *Jixueteng* extract inhibits osteoclast differentiation and survival in a dose-dependent manner, neutralizes oxygen species and improves blood flow, we developed a dentifrice containing *Jixueteng* in this study. However, *Jixueteng* alone did not have a satisfactory bactericidal effect on periodontopathic bacteria and fungi. Therefore, the antimicrobial effect was supplemented with a mastic, which effectively suppresses pathogenic bacteria. Furthermore, as a result of searching for plant-derived components that suppress periodontopathic bacteria, we found that antibacterial lotuses had comprehensive and effective antibacterial effects on pathogenic bacteria and fungi. We are commercializing a dentifrice containing these ingredients and named it "IMPLA CARE" (Figure 19).

At our hospital, dental hygienists do not invasively remove dental calculus in primary care. Patients were supplied with an IMPLA CARE at night-time and the dental calculus was removed after the gums were healthy and tight. Improving gingiva before treatment with IMPLA CARE reduced the risk of bacteraemia. IMPLA CARE was important for subsequent treatment, postoperative management and may help prevent systemic diseases such as diabetes, cerebral infarction and myocardial infarction. We investigated natural products with antimicrobial activity. First, the minimum inhibitory concentrations (MIC) of *Jixueteng*, *Sasa veitchii* and lotus on oral bacteria were measured. The natural products were dissolved in sterilized phosphate-buffered saline (PBS; pH 7.4) and two-fold serial dilutions were aliquoted in small volumes in microwell plates. Bacterial cells were grown in brain heart infusion (BHI) broth supplemented with hemin (5 µg/mL), vitamin K_1_ (0.2 µg/mL) and yeast extract (5 mg/mL) under anaerobic conditions (CO_2_: 10%, H_2_: 10%, N_2_: 80%) at 37°C for 18 h. Bacterial cells were washed and suspended in PBS to an optical density of 1.0 at 600 nm. The bacterial suspension was exposed for 40 h to two-fold serial dilutions of the natural products. The same volume of PBS was used as a control. *Jixueteng* had bactericidal effects on *S. mutans, L. casei, S. gordonii, F. nucleatum* and *S. aureus*. *Sasa veitchii* had a strong bactericidal effect on the fungus *C. albicans* (Table 9). Therefore, the IMPLA CARE contained a mixture of *Jixueteng*, *Sasa veitchii*, grapefruit and lotus in the mastic resin.

**Figure 21:**
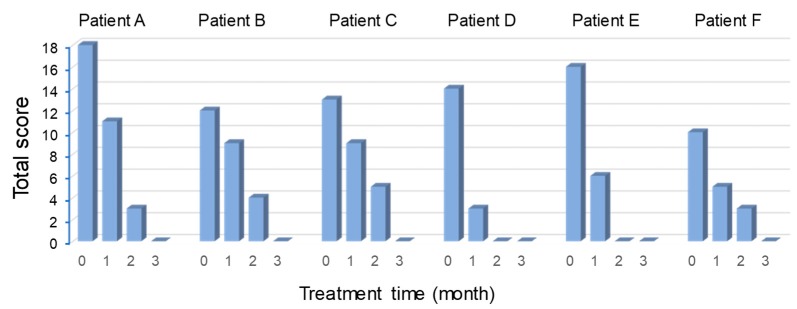
Effects of the IMPLA CARE. (Watanabe S, unpublished data)

Six patients with periodontal disease, peri-implant inflammation or both were examined. Results were measured after using IMPLA CARE and 1–3 times daily tooth brushing and 1–3 months of light massage of the affected part with fingers (Table 9 and Table 10). Periodontal disease improved in all patients (Table 11, Figure 21).

**Figure 22:**
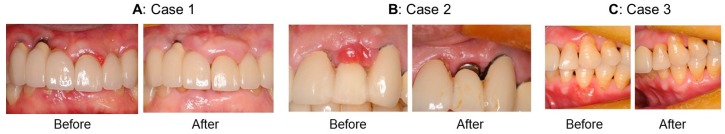
Case 1: A patient who was developing diabetes and hypertension (**A**), Case 2: A patient with peri-implantitis (**B**), Case3: A patient with an ulcer from sleep deprivation and work stress (**C**). (Suzuki M, unpublished data)

We evaluated patients who were not examined by dentists and found a self-reported improvement (Figure 22). Patient satisfaction also increased, which suggests that IMPLA CARE can be used as a primary care tool after treatment.

Case 1: A patient who was developing diabetes and hypertension. The patient had an implant in the anterior teeth of the maxilla and had teeth cleaned regularly at a dental clinic but gingivitis persisted. The doctor instructed the patient to use the IMPLA CARE before going to bed every night. Gingivitis improved gradually (Figure 22A).

Case 2: A patient with peri-implantitis. After insertion of an implant at 65 years old, a secondary operation was performed but peri-implantitis developed after the second operation. The doctor instructed the patient to use the IMPLA CARE himself before going to bed every night. Conditions gradually improved and a superstructure was formed after one month (Figure 22B).

Case3: A patient with an ulcer from sleep deprivation and work stress. A patient developed an ulcer from work stress and sleep deprivation. An IMPLA CARE was used daily in the morning and evening and improvements were observed with a week, which suggests that the IMPLA CARE containing traditional Chinese medicines did not cause gingival recession (Figure 22C).

## 8. Conclusions and Future Studies 

Kampo is a historic traditional medicine that has been adjusted to Japanese culture. The concept of Kampo emphasizes the relationship between the human body and its social and natural environments [2]. Our experiments concluded that Kampo (JTX and *Jixueteng*) reduce a great effect on oral bacteria and inhibited the bacteria-induced alveolar bone loss. Kampo also suppressed the osteoclast differentiation. Furthermore, Kampo improved the inflammatory response in the periodontal tissues of patients. These findings suggest that Kampo is an effective agent for the prevention of dental caries and periodontitis. The administration of Kampo may ameliorate to infected oral tissue environment.

Oral health is related to life-style such as diet in many ways. The development of dental caries requires high sugar intakes [62]. On the other hand, the high consumptions of smoking and alcohol and the loss of vitamin D affects metabolic functions of periodontal tissue and induce periodontitis. Previous review has been reported that psychological stress reduces human immune system and promotes chronic inflammation in periodontal tissue [63]. Our result demonstrated that JTX affected the correlation between restraint stress and bacteria-induced periodontal destruction [6,14]. Recently, psychological stress is a risk factor of toothache, especially non-odontogenic pain [64]. Odontogenic pain is generally derived from pulpal or periodontal tissue. However, non-odontogenic pain is not often originated from the orofacial regions. The characteristics of non-odontogenic pain indicate various types of symptoms; very mild, intermittent and severe, sharp pain and continuous. The general dentists are difficult to be specified the pain regions, that confuse the exact pain control in any case. In the clinical suggestion of effective pain control, the use of Kampo is expected to reduce the non-odontogenic pain [65]. In the future, the mixed concept of Western medicine and Kampo medicine will contribute in the treatment and prevention of several oral diseases.

**Figure 23:**
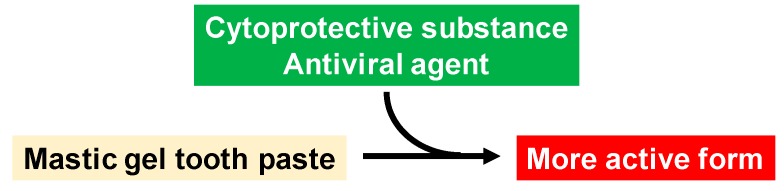
Manufacturing of advanced mastic gel tooth paste.

Supplementation of alkaline extract of *Sasa sp*. leaves (SE), which can alleviate the deoxorubicin-induced keratinocyte cytotoxicity [66] and paclitaxel-induced neurotoxicity [67] by promoting hermetic cell growth. and have anti-HIV activity [68], may enhance the potential of mastic gel tooth paste (Figure 23).

## Figures and Tables

**Table 1:** Definitions and classifications of *Yin-Yong*, *Kyo-Jitsu* and *Kan-Netsu*.

Categories	*Yin-Yang*	*Kyo-Jitsu* (Asthenia and Sthenia)	*Kan-Netsu* (Chills and Fever)
Definitions	Ability to resist the disease. The treatment differs, depending on whether the *sho* is *yang* and *yin*. *Yin* disease: The resistant power against the disease is weak. Treatment is given to recover the exhaustion of the body. It is called *honchi* or the systemic meridian treatment	The state of deficiency is *kyo-sho* or asthenia and excess is *jitsu-sho* or sthenia. *Kyo* (Asthenia): Deficiency of what is required for the body and shows the *sho* of *Yin-kyo* syndrome or *yang-kyo* syndrome	Actual feeling of chill and heat *Kyo-netsu* (asthenic heat): Heat coming from poor metabolism and retained inside the body, slight fever, internal fever, showing *yin*-syndrome.
Classification (According to the Orthodox Theory)	*Yang* disease: The resistant power against the disease is strong. Treatment which positivity attacks the disease is effective. It is called *hyochi* or local and symptomatic treatment.	*Jitsu* (Sthenia): Low metabolic rate due to the accumulation of toxic substance in the body and shows the *sho* of *yin-jitsu* or *yang-jitsu*.	*Jitsu-netsu* (sthenic heat): Chills that occurs by increasing the heat in the body. Observes pyrexia and showing *Yang*-syndrome.

**Table 2:** Definitions of *Yin-Kyo*, *Yo-Kyo*, *Yin-Jitsu* and *Yo-Jitsu*.

Categories	*Yin-Kyo* (*yin* deficiency)	*Yo-Kyo* (*yang* deficiency)	*Yin-Jitsu* (*Yin* excess)	*Yo*-*Jitsu* (*Yang* excess)
Definitions	*Yin* is insufficient and *yan* becomes relatively overactive; shows fever called asthenic fever; feverish symptom.	*Yang* is insufficient and *yin* relatively becomes overactive; asthenia cold; feeling chills.	Coldness comes from outside to constantly maintain the homeostasis of *yin* and *yang* and shows the state of chills, called *Jitsu-Kan* or sthenic cold; body is chilled and feeling cold.	Heat comes from outside to constantly maintain the homeostasis of *yin* and *yang*; and shows the state of heat, called *Jitsu-Netsu* or sthenic heat; feeling hot and excessive sw eating.

**Table 3:** Classifications of *Ki*, *Ketsu* and *Sui*.

Yin	Yang
Ketsu Sui	Ki

**Table 4:** The correlations between the colours and shapes in tongue.

Colour Tone	***Pale tongue:*** Paler colour than normal tongue; showing deficiency of *Ki* and *Ketsu*. ***Reddish tongue:****Netsu-Sho* (heat) symptom; redder than normal tongue colour and often shows dryness of mouth and lips. ***Purplish tongue:*** Systemic *Oketsu* due to stagnation of *Ki and Ketsu;* especially it is very likely when the dorsal lingual veins are over swelling. If the tongue is wet, it is *Kan-Sho* (cold) symptom and the body is chilled. If dry, it shows *Netsu-Sho*.
Shape	***Swelled tongue:*** Tongue is swollen and shows tooth marks on peripheral; showing *Ki-Kyo* and S*ui-Tai* (deficiency of *Ki* and fluid stagnation). ***Thin tongue:*** Tongue is dry and in deep purple colour; shows *Sui-Kyo, Yin-Yo-Kyo* (deficiency of *Sui, Kyo* of both *Yin and Yang.* ***Trembling tongue:*** When moving the tongue, it trembles; it vibrates; showing *Netsu-Sho* and *Ki-Kyo (Ki* deficiency).

**Table 5:** The correlations between the colours and volumes in tongue.

Colour Tone	***White tongue coating****:* Pale white colour is considered normal but shows the changes in body condition, depending on the wetness ***Yellow tongue coating:*** Shows stomach/intestine function deficiency; fever; lack of water due to fever ***Greyish black tongue coating****:* Yellow tongue coating symptom progressed and disease worsened; related to infectious disease, high fever, dehydration
Volume	***Thin tongue coating****:* Normal ***No tongue coating****:* Abnormal; chronical disease; protracted illness ***Thick tongue coating****:* Abnormal; exacerbation of disease, growth of bacteria due to defective metabolism of tongue coating

**Table 6:** Effects of *Jixueten*g on osteoclast formation in periodontal tissues.

Groups	Number of Osteoclasts		
	2W	4W	6W
Control	14.25 ± 1.71	16.00 ± 1.00	10.33 ± 0.58
*P. gingivalis*	18.50 ± 5.45	29.00 ± 8.25 **	23.33 ±1.53 **
*Jixueteng* + *P. gingivalis*	15.75 ± 7.41	17.00 ± 2.94	12.33 ± 3.21

** Significantly different (*p* < 0.01) from Group A and C. The number of osteoclasts was examined in the section from right maxillary specimen stained of tartrate-resistant acid phosphatase. The results were expressed as mean ± standard deviation. Group A, control (non- infected with *P. gingivalis*); group B, orally infected with *P. gingivalis*; group C, administered *Jixueteng* and orally infected *with P. gingivalis*. Cited from [28] with permission.

**Table 7:** Minimum inhibitory concentration (MIC) of mastic resin oil. (Hamada N, unpublished data).

Oral Bacteria	MIC (%)
*Streptococcus mutans*	0.4
*Streptococcus sanguinis*	0.4
*Streptococcus mitis*	0.4
*Lactobacillus species*	0.2
*Staphylococcus aureus*	0.8
*Bacillus species*	0.2
*Actinomyces species*	0.2
*Porphyromonas gingivalis*	< 0.05
*Porphyromonas endodontalis*	1.6
*Prevotella intermedia*	1.6
*Fusobacterium nucleatum*	< 0.05
*Aggregatibacter actinomycetemcomitans*	0.2

**Table 8:** Plaque formation on the tooth surface and effect of inhibition of gingivitis of mastic gums. (Hamada N, unpublished data).

Group	Plaque Index		Gingivitis Index	
	Baseline	1 week	Baseline	1 week
Mastic gum (n = 10)	1.06 ± 0.29	2.69 ± 0.29 **	0	0.44 ± 0.15*
Placebo gum (n = 10)	1.19 ± 0.19	3.15 ± 0.24 **	0	0.66 ± 0.23*

These data are represented as mean ± standard deviation. No statistical difference was observed between the groups at baseline. *p* < 0.05* *p* < 0.001** comparison with the baseline using Student’ test. The data are determined as the lowest concentrations of mastic resin oil.

**Table 9:** Minimum inhibitory concentration (MIC) of five natural products. (Hamada N, unpublished data).

Tested bacteria	*Jixueteng*	Sasa Veitchii	Grapefruit	Propolis	Lotus
*Streptococcus mutans*	4	> 8192	512	256	8192
*Streptococcus gordonii*	8	4	2048	8192	> 8192
*Lactobacillus casei*	4	> 8192	512	256	8192
*Staphylococcus aureus*	32	> 8192	4096	> 8192	> 8192
*Actinomyces viscosus*	32	4	4096	256	8192
*Porphyromonas gingivalis*	> 8192	512	4096	> 8192	> 8192
*Prevotella nigresecens*	> 8192	128	> 8192	> 8192	> 8192
*Fusobacterium nucleatum*	16	8	4096	2048	> 8192
*Escherichia coli*	> 8192	> 8192	1024	16	8192
*Candida albicans*	64	8	8192	2048	> 8192

**Table 10:** Scores showing the progress of periodontal disease.

Examination Criteria							
Score	Swollen Pus	Redness	Bleeding	Pus Discharge	Gingival Colour	Mobility	Patient’s Opinion
5	Papilla and adhering to gingiva	Extending to the papilla and gingival gums	Naturally bleeding	Naturally draining	Dark red purple	Upper and lower lip and tongue immobile	No change
3	Papilla and extending to the gingival margin	Papilla and tooth inflammation	Bleeding by acupressure	Acupressure-induced draining	Dark red	Strongly immobile	Improved a little
1	Part of the papilla	Part of the papilla	Slight bleeding with acupressure	Slight draining with acupressure	Brilliant	Slightly immobile	Tightened
0	None at all	No redness	None at all	No discharge at all	Light pink	Within a physiological range	Improved a lot

**Table 11:** Improvement of periodontal disease after administration of the IMPLA CARE. (Watanabe S, unpublished data).

Examination Items	Scores at 0, 1, 2 or 3 Months Later																							
	Patient A				Patient B				Patient C				Patient D				Patient E				Patient F			
	0	1	2	3	0	1	2	3	0	1	2	3	0	1	2	3	0	1	2	3	0	1	2	3
Swollen pus	1	0	0	0	1	0	0	0	3	2	1	0	3	0	0	0	5	3	0	0	1	1	0	0
Redness	5	3	1	0	3	2	1	0	3	2	1	0	3	0	0	0	3	1	0	0	3	1	1	0
Bleeding	5	3	1	0	3	2	1	0	2	1	1	0	2	0	0	0	3	0	0	0	1	0	0	0
Pus discharge	1	0	0	0	1	0	0	0	3	2	1	0	3	0	0	0	1	0	0	0	1	0	0	0
Gingival colour	3	1	0	0	3	2	1	0	2	1	1	0	2	3	0	0	3	1	0	0	3	1	1	0
Mobility	3	1	0	0	1	0	0	0	0	0	0	0	0	0	0	0	1	0	0	0	1	1	1	0
Patient’s opinion		3	1	0		3	1	0		1	0	0		0	0	0		1	0	0		1	0	0
Total score	18	11	3	0	12	9	4	0	13	9	5	0	14	3	0	0	16	6	0	0	10	5	3	0

## Abbreviations

ABC	alveolar bone crest
CC_50_	50% cytotoxic concentrations
CEJ	cemento-enamel junction
CFU	colony forming unit
CMC	carboxymethylcellulose
COX	cyclooxygenase
CYP	cytochrome P450
EC_50_	50% effective concentration
FBS	foetal bovine serum
GC/M	gas chromatography-mass spectrometry
GRS	goreisan
HGF	human gingival fibroblast
HIV	human immunodeficiency virus
HPC	human pulp cell
HPLF	human periodontal ligament fibroblast
HSV	herpes simplex virus
IC_50_	50% inhibitory concentration
kD	kilo dalton
LPS	lipopolysaccharide
MIC	minimum inhibitory concentration
NO	nitric oxide
PGE_2_	prostaglandin E_2_
RANKL	receptor activator of NF-κB ligand
RCB	red complex bacteria
SI	selectivity index, determined by the ratio of CC_50_/EC_50_
TNF	tumour necrosis factor
TRAP	tartrate-resistant acid phosphatase
TS	tumour specificity, determined by ratio of CC_50_ for normal cells to CC_50_ for tumour cells

## Appendix A

**Table A1:** Kampo terminology.

*Byo-ja*	Pathogen, disease inducing factor
*Ei-ki*	Ying energy, *Ki* which operates the blood flows in the blood vessels. Without *ki*, blood does not circulate
*Honchi*	Systemic meridian treatment for systemic route cause of the disease
*Hyo*	Superficies, Surface of the body
*Hyochi*	Local and symptomatic treatment for symptoms derived by the rout cause
*Hyo-sho*	Pathological condition which appears on the surface of the boy
*In*	*Yin*, the state opposite of *Yang*, including *ri*, *kan*, *kyo*, *shuren* (convergence) and *yukei* (tangible)
*In-Yo*	*Yin-Yang*, Bipolar nature of all phenomena or viewing the phenomena from bipolar aspect
*Ja*	Stress/pathogenic factor, disease-inducing factor that is harmful to the body; endogenous factor, exogenous factor, neither endogenous or exogenous factor
*Jitsu*	Excess of required substance of body being a disease inducing factor or its pathological condition
*Jitsu-kan*	Sthenic chill; *Kan-ja* inhibits *yang-ki* (heat energy) in body and deteriorates body functions
*Jitsu-netsu*	Sthenic heat/excess heat, affected by the exogenous cause of heat; or heat from dental stress and intemperance
*Jitsu-sho*	Robust/excess constitution, condition or characteristics of over-reaction caused by *byo-ja*
*Kan*	Chill, symptom showing coldness
*Kan-netsu*	Chills and fever, pathological condition of chill and heat
*Kan-sho*	*Kan-sho*, pathological condition showing chilly feeling
*Kekkyo*	*Ketsu-kyo*, pathological condition of *ketsu* insufficiency
*Ketsu-netsu*	Blood-heat, *ketsu* (blood) affected by heat-ja and shows *kyo-netsu*
*Ki*	Vital energy that operates body functions
*Ki-gyaku*	Pathological condition of *ki* regression
*Ki-kyo*	*Ki* deficiency, state of insufficient or deficient *ki*
*Ki-kyo*	*Ki* deficiency, state of insufficient or deficient *ki*
*Ki-tai*	*Ki* stagnation, pathological condition of *ki* stagnation
*Ko-kan*	Xerostomia, feeling mouth dryness but not wanting to drink water; tends to occur with mental strain
*Ko-katsu*	Mouth dryness, feeling thirst and wanting to drink water
*Kyo*	Deficiency/asthenia, insufficiency of functions or physiological substance that physical body requires
*Kyo-kan*	Asthenia-cold, *kyo-sho* symptom and belongs to Kan; insufficient *yang-ki* (heat) to warm up the body
*Kyo-netsu*	Asthenic heat-syndrome, where *yin-eki* is insufficient and relatively *yang* becomes overactive feeling feverish
*Kyo-sho*	asthenia constitution; Pathological condition of insufficiency of fundamental substances that operate body functions
*Nai-netsu*	Internal heat-syndrome, generated as a relative result of the imbalance of *yin-yang*; *jitsu-netsu*, *kyo-netsu*
*Okets*	Blood stagnation, symptom caused by the stagnation of the blood flow
*Ri*	Interior of the entire body
*Sansho*	a passage that controls the flow of air, blood and water, called “triple heater “
*Shin-eki*	Bodily transparent fluid which constitutes the human body
*Shitsu*	Dampness of morbidly sustained fluid in the body as a disease inducing factor
*Sho*	Kampo diagnosis, set of holistic pattern of a patient’s pathological symptoms that cause disease
*Shoko*	Kampo medical conditions, symptoms in Western medical terms
*Tan*	phlegm, sticky fluid locally pooled due to poor water metabolism
*Tan-in*	Diseases due to pathological accumulation of fluids in the body
Tongue coating	Mossy substance covering the surface of the tongue
*yang*	Chinese term for *Yo*
*Yin*	Chinese term for *In*
*Yin-eki*	Fluid consisting of human body and composed of transparent *shin-eki* and red blood
*Yang*	Yin-deficiency Symptom of heat due to insufficient Yin-eki
*Yo*	Yang, the state opposite of *Yin*, including *hyo*, *netsu*, *jitsu*, *hassan* (divergence) and *mukei* (intangible)
*Yo-kyo*	Yang-deficiency, pathological condition that chill of *ki-kyo* is worsened
*Yo-sho*	Yang-sho, condition that has characteristics of excitement, activity and warm-heat
*Shokan-ron*	A treatise on Shang han, a form of an acute infectious disease
